# A Clinical Risk Prediction Model for Identifying Patient Candidates for Same-day Discharge After Transcatheter Aortic Valve Replacement

**DOI:** 10.1016/j.jscai.2025.104110

**Published:** 2026-01-13

**Authors:** Asa Phichaphop, Vinayak N. Bapat, Nadira Hamid, Ellen Cravero, Larissa I. Stanberry, Rebecca Uelmen, Atsushi Okada, Miho Fukui, Hideki Koike, Davide Margonato, Cheng Wang, Maurice Enriquez-Sarano, João L. Cavalcante, John R. Lesser, Paul Sorajja

**Affiliations:** a Valve Science Center, Minneapolis Heart Institute Foundation, Minneapolis, Minnesota; b Division of Cardiology, Department of Medicine, Faculty of Medicine Siriraj Hospital Mahidol University, Bangkok, Thailand; c Minneapolis Heart Institute, Abbott Northwestern Hospital, Minneapolis, Minnesota; d Cardiovascular Imaging Research Center and Core Lab, Minneapolis Heart Institute Foundation, Minneapolis, Minnesota

**Keywords:** clinical risk prediction model, machine learning, same-day discharge, transcatheter aortic valve replacement

## Abstract

**Background:**

Same-day discharge after transcatheter aortic valve replacement (TAVR) may be feasible for selected patients if a low risk for adverse clinical events can be defined. We aimed to develop a clinical risk prediction model to facilitate same-day discharge planning.

**Methods:**

A random forest machine learning algorithm was used to build a prediction model of adverse events occurring in-hospital after TAVR. Patients were categorized into low, moderate, or high-risk groups based on their estimated scores.

**Results:**

Overall, 730 patients (median age, 81 years; 58.9% men) who had transfemoral TAVR performed with conscious sedation were examined. The risk score was built utilizing 9 clinical parameters. The prediction model had a median area under the receiver operating characteristic curve of 0.76. For determining the probability of events that would disallow same-day discharge, the model successfully identified 172 patients (23.6% of the population) as low-risk for same-day discharge, or for having an event rate of <3%, with all events occurring within 6 hours after TAVR. The low-risk group had no in-hospital events after a 6-hour observation, and no mortality at the 30-day follow-up. External testing in 158 patients showed 94% sensitivity in predicting overall adverse events and identified a low-risk group using the clinical risk score.

**Conclusions:**

In this analysis, ∼1 in 4 patients may be candidates for same-day discharge after TAVR. This prediction model can identify such patients, with findings that may have implications for hospital resource allocation in those undergoing TAVR.

## Introduction

For patients with severe, symptomatic aortic stenosis, transcatheter aortic valve replacement (TAVR) is a life-saving procedure. The number of patients being treated with TAVR continues to grow, with over 100,000 cases each year in the United States alone.^1^ These treatments create significant socioeconomic pressure, with a substantial amount of the cost burden falling on hospital systems of care.^2^

As a means of improving efficiency in care, the possibility of same-day discharge planning for patients undergoing TAVR has been raised. This importance becomes greater as the number of patients with relatively fewer morbidities (ie, low or intermediate surgical risk) who undergo the procedure increases.^3^, ^4^, ^5^ The implementation of prediction tools could help identify patients with low TAVR procedural risk, who could be scheduled early to facilitate same-day discharge following a period of clinical observation for adverse events. Although early studies have suggested several helpful clinical predictors, a machine learning algorithm may provide additional information for systematic and accurate predictors for identifying low-risk patients as candidates for same-day discharge.^6^^,^^7^

Accordingly, our primary aim was to develop a clinical risk prediction model incorporating clinical and imaging variables to identify TAVR patients with a low risk of adverse events. We focused on identifying adverse events occurring within the parameters of a program where a 6-hour postprocedural observation period is required, and the patient could then be safely discharged without a worsening impact on 30-day outcomes.

## Methods

### Study population

The study population consisted of patients evaluated and treated for aortic stenosis at Abbott Northwestern Hospital (Minneapolis, Minnesota) between January 2016 and December 2022. Inclusion criteria were: (1) severe, symptomatic aortic stenosis; (2) a heart team evaluation that determined clinical indication for TAVR; (3) anatomical suitability for transfemoral approach^8^; (4) eligibility for conscious sedation during the procedure; and (5) TAVR performed on the day of admission in an elective setting. Patients with a preexisting cardiovascular implantable electronic device (CIED) were excluded from the study.

The study population was divided into 2 cohorts. The development cohort, used for creating a prediction model with cross-validation, included patients from January 2016 to December 2021. The testing cohort, used for external testing, included patients from January 2022 to December 2022.

All patients provided informed consent for the utilization of their medical records for research objectives before their inclusion. The present investigation was approved by the Allina Institutional Review Board and was conducted in strict adherence to the principles of the Declaration of Helsinki.

### Clinical data and definitions

Baseline clinical characteristics, imaging data, and adverse events were extracted from the electronic medical records. The primary outcome was defined as the occurrence of any of the following adverse events during the index admission: atrioventricular block requiring permanent pacemaker, vascular complications, cardiac arrest, cardiovascular death, myocardial infarction, transient or disabling stroke, and cardiac perforation. Vascular complications included a major or minor complication related to the device insertion, delivery, and complete removal of all its components, and those requiring unplanned intervention or surgery. Stroke was defined as an acute onset of neurological signs and symptoms of any severity. All outcomes were aligned with VARC-3 definitions.^9^ The variables were extracted from the Society of Thoracic Surgeons/American College of Cardiology Transcatheter Valve Therapy (STS/ACC TVT) registry and the electronic medical records. Pre-TAVR computed tomography angiography was measured by an experienced imaging cardiologist as a part of routine TAVR care at our center. The measurements were used to define a minimal femoral artery diameter, adopted from the smallest arterial size of the cross-sectional planes, at the vessel selected for device introduction, with exclusion of calcification. Left ventricular outflow tract calcification was qualitatively graded as none, mild, moderate, or severe in accordance with standard definitions.^8^ These measurements were derived from routine assessments by cardiologists, documented in the electronic medical records as part of the TAVR heart team meeting. For patients treated with balloon-expandable prostheses, the percentage of valve area oversizing was calculated as follows: (TAVR valve nominal measurement/annular measurement – 1) × 100.

### Data analysis

Continuous variables were presented as mean ± SD or median with IQR and compared using the Wilcoxon rank-sum test. Categorical data were presented as counts (%) and compared using the χ^2^ or Fisher exact tests, as appropriate. Missing baseline characteristics were handled using complete case analysis. For insight into the relevance to a potential same-day discharge program and impact on 30-day outcomes, adverse events were also grouped according to those that occurred within the index admission, and those that occurred after hospital discharge within 30 days after TAVR. Events that occurred during the index admission were further classified as to whether they occurred within 6 hours of the TAVR procedure. This time period was chosen to reflect a duration that would be consistent with a postprocedural observation period in same-day discharge protocols.^5^^,^^10^^,^^11^

A random forest method was used to build a prediction model of clinical adverse events during the index admission. A total of 9 predictor variables (age, body mass index, history of myocardial infarction, significant coronary artery disease, preexisting right bundle branch block, left ventricular ejection fraction, left ventricular outflow tract calcification, minimal femoral artery diameter, valve type with oversizing percentage for balloon-expandable valve) were considered for the prediction model. Significant coronary artery disease was defined as ≥50% stenosis of left main or ≥70% stenosis of a non–left main major epicardial artery. We selected variables previously associated with risk of new pacemaker, vascular complications, stroke, annular rupture, and surgical bailout for the TAVR procedure.^12^, ^13^, ^14^, ^15^, ^16^, ^17^, ^18^, ^19^ Further variable selection was determined using machine learning feature ranking based on the reduction in mean accuracy, which ranks the predictors by the decrease in model accuracy if the variable is removed from the model. Further, we also included a random noise variable into the model and selected only features with higher importance than the noise variable.

The data were divided based on the outcomes distribution into training and testing sets at a ratio of 3:1. Model development was done on the training set using random forest with repeated cross-validation with 5 folds and 5 repeats, and model performance was assessed on the testing set. This process was repeated 10 times to make sure our findings were not a result of one specific training and testing split. Model performance metrics were summarized as median with IQR from the 10 different testing sets. The predictive performance of the model was estimated by the median area under the receiver operating characteristic curve (AUC) from the 10 repeats. Calibration of the model was assessed by the median calibration slope from the repeats.

We utilized the SHapley Additive exPlanations (SHAP) values to help reveal the specific relationship between each feature in the model and the final predicted risk, an element that is hidden in most machine learning models. A SHAP value can be thought of as the impact that a patient’s feature is making on the model output. We applied SHAP analysis to our final prediction model to provide a more comprehensive description of the impact each variable makes on the prognostic performance.

Furthermore, we defined 3 risk groups based on the estimated scores. The first threshold was defined by scores below the first quartile of the estimated scores from all patients. Patients in the low-risk group would be candidates for same-day discharge. The second threshold to separate moderate risk from high risk was chosen based on clinical expertise and aligned with the first threshold. In addition, we tested the performance of the model in a testing cohort and evaluated the model performance using AUC, as well as sensitivity and specificity for capturing the adverse events using the first threshold as a cutoff, and subsequently utilized both low and intermediate thresholds to demonstrate adverse events in the testing cohort.

All tests used a critical value of 0.05. All analyses were performed using R version 4.4.1 (R Core Development) in the RStudio 2024 (Posit Software, PBC) environment.

## Results

### Baseline characteristics

A total of 730 patients who underwent TAVR between January 2016 and December 2021 (median age, 81 years [IQR, 76-86]; 58.9% men) were included in the development cohort. Additionally, 158 patients who underwent TAVR between January 2022 and December 2022 met the study criteria and were included in the testing cohorts. The median hospital stay was 1 day (IQR, 1.0-2.0). Baseline characteristics, including comorbidity and variables for calculating the risk score, were summarized and compared between both development and testing cohorts (Table 1).

**Table 1:** Baseline patient characteristics.

Characteristics	Development cohort (n = 730)	Testing cohort (n = 158)	*P* value
Age, y	81.0 (76-86)	82 (77-86)	.63
Male sex	430 (58.9%)	90 (57.0%)	.74
Body mass index, kg/m^2^	29.0 (25.6-32.6)	28.2 (25.3-32.6)	.52
Diabetes	214 (29.3%)	42 (26.6%)	.58
Hypertension	612 (83.8%)	135 (85.4%)	.61
GFR <30 mL/min/1.73 m^2^	27 (3.7%)	7 (4.4%)	.76
Cerebrovascular disease	80 (11.0%)	23 (14.5%)	.69
Bicuspid aortic valve disease	49 ( 6.7%)	3 (1.9%)	.019
Bioprosthetic aortic valve	28 (3.8%)	5 (3.2%)	.73
Coronary artery disease history	318 (43.6%)	69 (43.7%)	.92
History of myocardial infarction	93 (12.7%)	26 (16.5%)	.27
Peripheral arterial disease	72 ( 9.9%)	17 (10.8%)	.75
Preexisting RBBB	92 (12.6%)	14 (8.9%)	.24
Preexisting LBBB	46 (6.3%)	13 (8.2%)	.45
Concomitant MR	97 (13.3%)	23 (14.6%)	.71
LVEF, %	62.3 (55-66)	63 (58-66)	.28
LVEF <40%	41 (5.6%)	11 (7.0%)	.56
Aortic valve area, mm^2^	0.80 (0.66-0.92)	0.84 (0.69-0.94)	.23
Moderate or severe LVOT calcification	72 (9.9%)	12 (7.6%)	.42
Balloon-expandable	505 (69.2%)	78 (49.4%)	<.001
Valve area oversizing,^a^ (%)	4.9 (-0.8 to 11.5)	2.0 (-1.7 to 7.0)	.011
Minimal femoral lumen diameter, mm	7.1 (6.4-8.0)	7.0 (6.0-8.0)	.48
Length of stay, d	1.0 (1.0-2.0)	1.0 (1.0-1.0)	—
STS-PROM score, %	2.8 (1.9-4.2)	2.5 (1.8-3.8)	.11

Values are median (IQR) or n (%).

GFR, glomerular filtration rate; LBBB, left bundle branch block; LVEF, left ventricular ejection fraction; LVOT, left ventricular outflow tract; MR, mitral regurgitation (at severity of moderate or severe); RBBB, right bundle branch block; STS-PROM, Society of Thoracic Surgeons Predicted Risk of Mortality.

a Valve area oversizing was calculated only in balloon-expandable valve use in patients.

### Clinical events

Overall, the composite outcome occurred in 91 (12.5%) patients of the development cohort and 18 (11.4%) patients in the external testing cohort. The most common adverse event was atrioventricular block requiring a permanent pacemaker and vascular complications in both groups. All events were observed during the index admission (Table 2).

**Table 2:** Adverse events occurred during index admission.

Adverse events	Development cohort (n = 730)	Testing cohort (n = 158)
All adverse events	91 (12.5%)	18 (11.4%)
AV block requiring permanent pacemaker	66 (9.0%)	12 (7.6%)
Vascular complications	17 (2.3%)	6 (3.8%)
Stroke	9 (1.2%)	1 (0.6%)
Cardiac perforation due to annular rupture	2 (0.3%)	2 (1.3%)
Cardiac arrest with successful resuscitation	1 (0.1%)	1 (0.6%)
Myocardial infarction	1 (0.1%)	0 (0%)
Cardiovascular death	0 (0%)	0 (0%)

Values are n (%).

AV, atrioventricular.

### Model development

**Figure 1:**
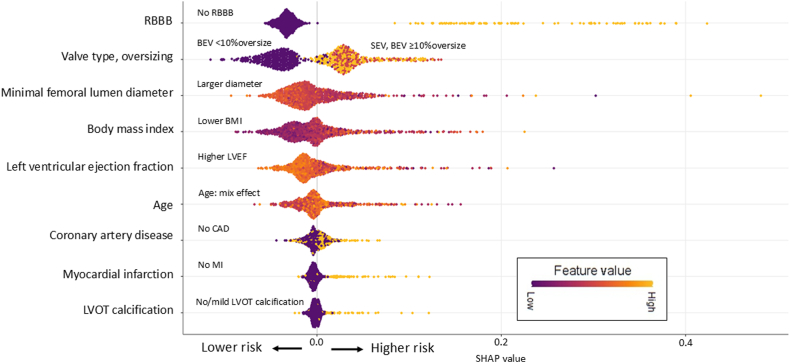
**Relationship of variables in the model illustrated by Shapley Additive exPlanations (SHAP) value.** The variables of importance in terms of relative weight on the model’s predictions and SHAP value are shown in order of variable importance. A feature with a higher SHAP value results in a higher risk of outcomes for the patients. For continuous variables, darker colors represent lower values and lighter colors represent higher values of each feature. BEV, balloon-expandable valve; BMI, body mass index; CAD, coronary artery disease; LVEF, left ventricular ejection fraction; LVOT, left ventricular outflow tract; MI, myocardial infarction; RBBB, right bundle branch block; SEV, self-expandable valve.

**Figure 2:**
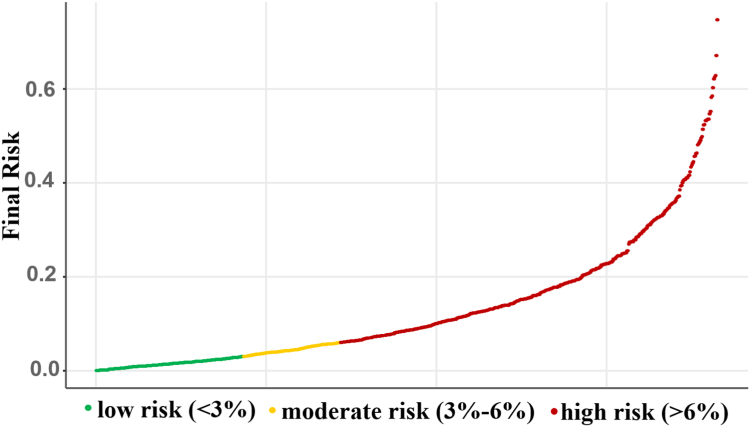
**Patient classification by estimated clinical risk score.** Patients were classified into low (<3%), moderate (3%-6%), and high (>6%) risk groups using the estimated risk score.

A clinical risk prediction model was built using 9 variables from the development cohort. The mean decrease in accuracy for all 9 variables in the model exceeded that of a random noise factor. The variables of importance in terms of relative weight on the model’s predictions and SHAP value are shown (Figure 1). The most important feature was right bundle branch block, with its presence largely increasing the risk of the outcome. The risk score for the individual patient was calculated and classified into 3 risk groups (Figure 2).

### Model performance and clinical applicability

The clinical risk prediction model yielded a good discrimination for adverse events with a median AUC of 0.76 (IQR, 0.73-0.77) across repeated validation (Central Illustration). The median calibration slope was 0.92 (IQR, 0.77-1.10). The model was particularly well calibrated for patients with lower risk (Supplemental Figure S1).

**Central Illustration:**
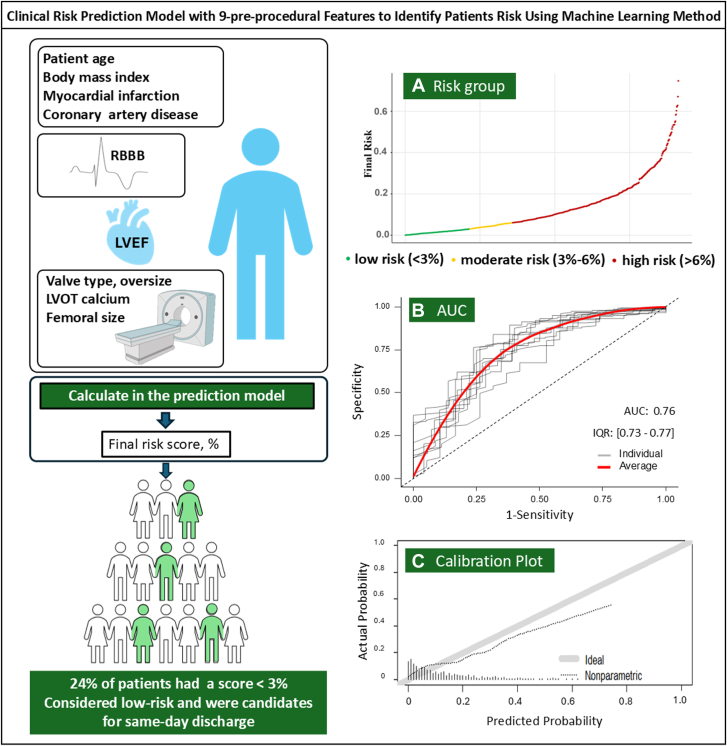
**Clinical risk prediction model with 9 preprocedural features to identify patients' risk using a machine learning method.** A risk score calculated from the prediction model with 9 preprocedural features (upper left box). Patients with low risk will have the best chance of same-day discharge prediction. (A) Clinical risk scores were demonstrated in low-, moderate-, and high-risk groups. (B) The ROC curve showed good predictive performance of the machine learning model with a median area under the receiver operating characteristic curve (AUC) of 0.76 (red line). (C) A calibration plot demonstrated that risk scores were well calibrated for low-risk patients, aligning closely with the ideal line (gray color). IQR, interquartile range; LVEF, left ventricular ejection fraction; LVOT, left ventricular outflow tract; RBBB, right bundle branch block; ROC, receiver operating characteristic curve.

**Figure 3:**
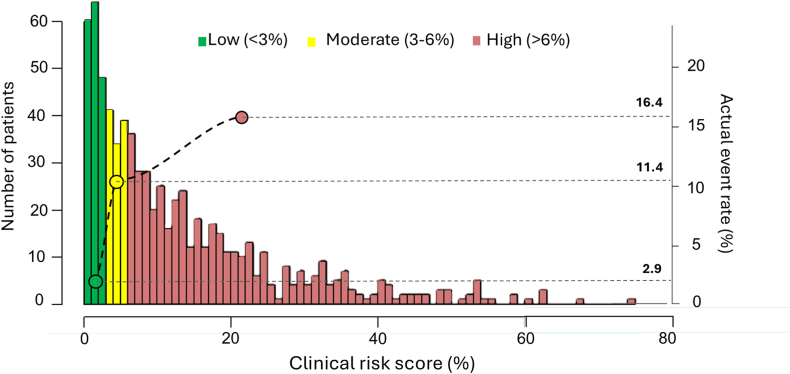
**Clinical risk score correlation with adverse events.** Patients with calculated clinical risk score demonstrated in a histogram and classified into low-, moderate-, and high-risk (green, yellow, and pink bars, respectively). The actual event rate in each group is shown.

For the development cohort, the median risk score was 8.6% (IQR, 3.3%-13%). Patients were grouped into categories of low risk (<3%), moderate risk (3%-6%), and high risk (>6%), leading to identification of 172, 114, and 444 patients, respectively. The actual observed adverse event rates in-hospital were 2.9%, 11.4%, and 16.4% (overall *P* < .001) for each of these risk groups, respectively (Figure 3, Supplemental Figure S2). Overall, most of the adverse events (83 events, 91%) were detected within the first 6 hours. Importantly, for the patients identified as low-risk in the predictive model, all adverse events occurred within the first 6 hours (Supplemental Table S1).

The testing cohort consisted of 158 patients with comparable baseline characteristics compared to the development cohort. Twenty-two percent of patients in this cohort had a clinical risk score <3% and were classified as low risk, with an adverse event rate of 2.9%, whereas patients with a clinical risk score ≥3% had an adverse event rate of 13.8%; particularly, intermediate risk group had an adverse event rate of 12%, and high-risk group had an adverse event rate of 14.3%. Utilizing this 3% cut point provided a 94% sensitivity for detecting overall adverse events which occurred in non–low-risk patients (Supplemental Table S2). Additionally, stratifying the testing cohort into low-, intermediate-, and high-risk groups demonstrated a progressively higher event rate with increasing risk category (Supplemental Table S3).

The incidences of 30-day postdischarge adverse events in the development cohort were similar across the 3 risk groups for all components of the composite outcomes. Furthermore, the 30-day cardiovascular readmission was similar between the risk groups (Table 3). For adverse events between 6 hours after TAVR to the 30-day follow-up, the incidence rates were similar among the 3 risk groups (low vs moderate vs high; 4.7% vs 7.9% vs 5.9%, *P* = .52).

**Table 3:** Adverse events in the development cohort occurring after hospital discharge within 30 days.

Adverse events	Low risk (n = 172)	Moderate risk (n = 114)	High risk (n = 444)	*P* value
Overall adverse event rate	8 (4.7%)	8 (7.0%)	21 (4.7%)	.59
AV block requiring CIED	4 (2.3%)	2 (1.8%)	10 (2.3%)	.94
Vascular complications	0 (0%)	1 (0.9%)	5 (1.1%)	.38
Stroke	4 (2.3%)	4 (3.5%)	5 (1.1%)	.19
Death	0 (0%)	1 (0.9%)	0 (0%)	>.99
Aortic reintervention	0 (0%)	0 (0%)	1 (0.2%)	>.99
Pericardiocentesis	1 (0.6%)	0 (0%)	1 (0.2%)	>.99
Myocardial infarction	0 (0%)	0 (0%)	1 (0.2%)	>.99
Any-cause readmission	16 (9.3%)	13 (11.4%)	42 (9.5%)	.80
Cardiovascular readmission	12 (7.0%)	12 (10.5%)	35 (7.9%)	.54
Procedure/valve-related	11 (6.4%)	12 (10.5%)	29 (6.5%)	.31
Bioprosthetic valve dysfunction	0 (0%)	0 (0%)	0 (0%)	—
Bleeding	1 (0.6%)	2 (1.8%)	2 (0.5%)	.32
Heart failure	0 (0%)	1 (0.9%)	7 (1.6%)	.23
Other cardiovascular reasons	1 (0.6%)	0 (0%)	6 (1.4%)	.35

Values are n (%).

AV, atrioventricular; CIED, cardiac implantable electronic device.

## Discussion

To the best of our knowledge, this clinical risk prediction model is the first prediction tool to identify TAVR patients who may be safely discharged on the day of the procedure. In this analysis, we found that ∼1 in 4 patients may be such candidates. These patients, identified as low-risk, exhibited an infrequent incidence of in-hospital adverse events (2.9%), with all events occurring within 6 hours of the procedure. Notably, the event rate was similar across low-, moderate-, and high-risk groups between 6 hours and 30 days after TAVR, demonstrating that an early discharge was not associated with patient harm.

In our study, we used a novel approach with a clinical risk prediction model that was based on machine learning algorithms with a random forest method. These algorithms estimate the risk of outcome across an ensemble of models, allowing an accurate prediction. A total of 9 practical clinical parameters yielded a strong prediction result. The clinical risk prediction model was then used to identify patients at low risk (score <3%) of adverse events post-TAVR and thus suitable for same-day discharge. The model demonstrated strong discrimination with a median AUC of 0.76 and was well calibrated for the low-risk patients. The model also performed well in the external testing cohort for the risk threshold of 3%. We were able to use this estimated score to achieve a sensitivity of 94%, which supports our main purpose that the prediction model correctly identifies the at-risk patients. We demonstrated a higher event rate in the intermediate and high-risk groups compared to the low-risk group in both the developing and testing cohorts. Our preprocedural risk model leverages routinely collected clinical variables in clinical practice. With a heart team approach, this methodology could add more information in decision-making processes for patient care, with the ultimate goal of optimizing early discharge without compromising patient safety.^10^^,^^11^

As described for other catheter-based cardiovascular procedures, same-day discharge is generally known to reduce the risk of hospitalization-related events, such as nosocomial infection, delirium, and deconditioning. Although our clinical risk prediction model is intended to be practical for identifying a low-risk group, the cut point of adverse events could be adjusted based on individual institutional preferences. Importantly, consideration of same-day discharge protocols should be carried out only in the context of appropriate family support and proper postdischarge care, including implementing routine next-day follow-up.

### Limitations

The study limitations include the retrospective, single-center design with predominantly low Society of Thoracic Surgeons scores, fewer comorbidities and preserved LVEF, and limited sample size. To counteract the latter limitation, we used the multisplit approach with cross-validation at each split as well as the random forest algorithm, which is one of the most accurate classifiers available in machine learning. Our factor selection was restricted to variables with established evidence of association with adverse events. However, this approach limited our ability to identify novel risk factors that may be specific to our patient population. The second threshold in our study was included for methodology consistency, but demonstrated compromised calibration, and larger studies may be needed to validate this threshold. We restricted our analyses to patients without preexisting CIED leads to minimize their presence or absence as a confounder, because patients with preexisting pacemakers require separate analysis, and conduction system issues are not a primary concern in this population.

## Conclusion

Our clinical risk score identifies patients undergoing transfemoral TAVR who may be suitable for same-day discharge as a pilot study. A further study to examine the broad capability of this approach is needed.

## Declaration of competing interest

Paul Sorajja has received consulting fees from 4C Medical, Abbott Structural, Adona, Boston Scientific, Edwards Lifesciences, EvolutionMedVentures, Foldax, GE Medical, Laza, Medtronic, Philips, vDyne, and xDot. Vinayak N. Bapat has received consulting fees from Abbott Structural, Anteris, Boston Scientific, Edwards Lifesciences, and Medtronic. Nadira Hamid has received consulting fees from Abbott Structural, Anteris, AMX, 4C Medical Technologies, Alleviant Medical, Edwards Lifesciences, Philips, GE, Valcare Med Ltd, Vdyne, and WL Gore. Miho Fukui has received consulting fees from Anteris and Edwards Lifesciences. Maurice Enriquez-Sarano has received consulting fees from Cryolife, Edwards Lifescience, Highlife, and ChemImage. João L. Cavalcante has received consulting fees from 4C Medical, Abbott Structural, Alleviant, Anteris, Boston Scientific, Circle Cardiovascular Imaging, Edwards Lifesciences, JenaValve, JC Medical, Medtronic, Novo Nordisk, Pie Medical, Siemens Healthineers, Shockwave Medical, and Zoll.
