# Supplementary material for: A Clinical Risk Prediction Model for Identifying Patient Candidates for Same-day Discharge After Transcatheter Aortic Valve Replacement
Source: J Soc Cardiovasc Angiogr Interv. 2026 Jan 13;5(2):104110. doi: 10.1016/j.jscai.2025.104110 (PMC12923339; doi:10.1016/j.jscai.2025.104110)
Supplement: Supplementary Material [file mmc1.docx]

**Supplemental Table S1** **Overall adverse events classified by time period and risk score in development cohort**

|  | Low risk  (n=172) | Moderate risk (n=114) | High risk (n=444) | p-value |
| --- | --- | --- | --- | --- |
| First 6 hours | 5 (2.9) | 12 (10.5) | 66 (14.9) | <0.001 |
| From 6 hours to discharge | 0 (0) | 1 (0.9) | 7 (1.6) | 0.23 |
| 30 days post-discharged events | 8 (4.7) | 8 (7.0) | 21 (4.7) | 0.58 |
| From 6 hours to 30 days | 8 (4.7) | 9 (7.9) | 26 (5.9) | 0.52 |

Patients in low risk group had a lower first 6 hours and didn’t have any adverse event from 6 hours to discharge. Adverse events at 30 day follow-up were not difference among groups.

**Supplemental Table S2 Sensitivity and specificity of detecting adverse events using clinical risk score in testing cohorts**

|  | **Adverse event**  **Yes** | **Adverse event**  **No** |  |
| --- | --- | --- | --- |
| Estimated risk score ≥ 3% | 17 | 106 | **PPV**  **14%** |
| Estimated risk score < 3% | 1 | 34 | **NPV**  **97%** |
|  | **Sensitivity**  **94%** | **Specificity**  **24%** |  |

Assess the performance of the estimated risk score in testing cohorts with a threshold of 3% demonstrated high sensitivity and high negative predictive value. PPV = positive predictive value; NPV = negative predictive value. Implement ≥ 3% cutoff can correctly identify 94% of adverse events (17 out of 18).

**Supplemental Table S3 Adverse events in a testing cohort stratified into low-, intermediate, high risk groups (**number of adverse events / total patients in the group (%))

| **Overall** | **Low-risk** | **Intermediate-risk** | **High-risk** |
| --- | --- | --- | --- |
| 18/158 (11.4%) | 1/34 (2.9%) | 3/25 (12%) | 14/98 (14.3%) |

**Supplemental Figure S1 - Calibration plot and AUC curve**


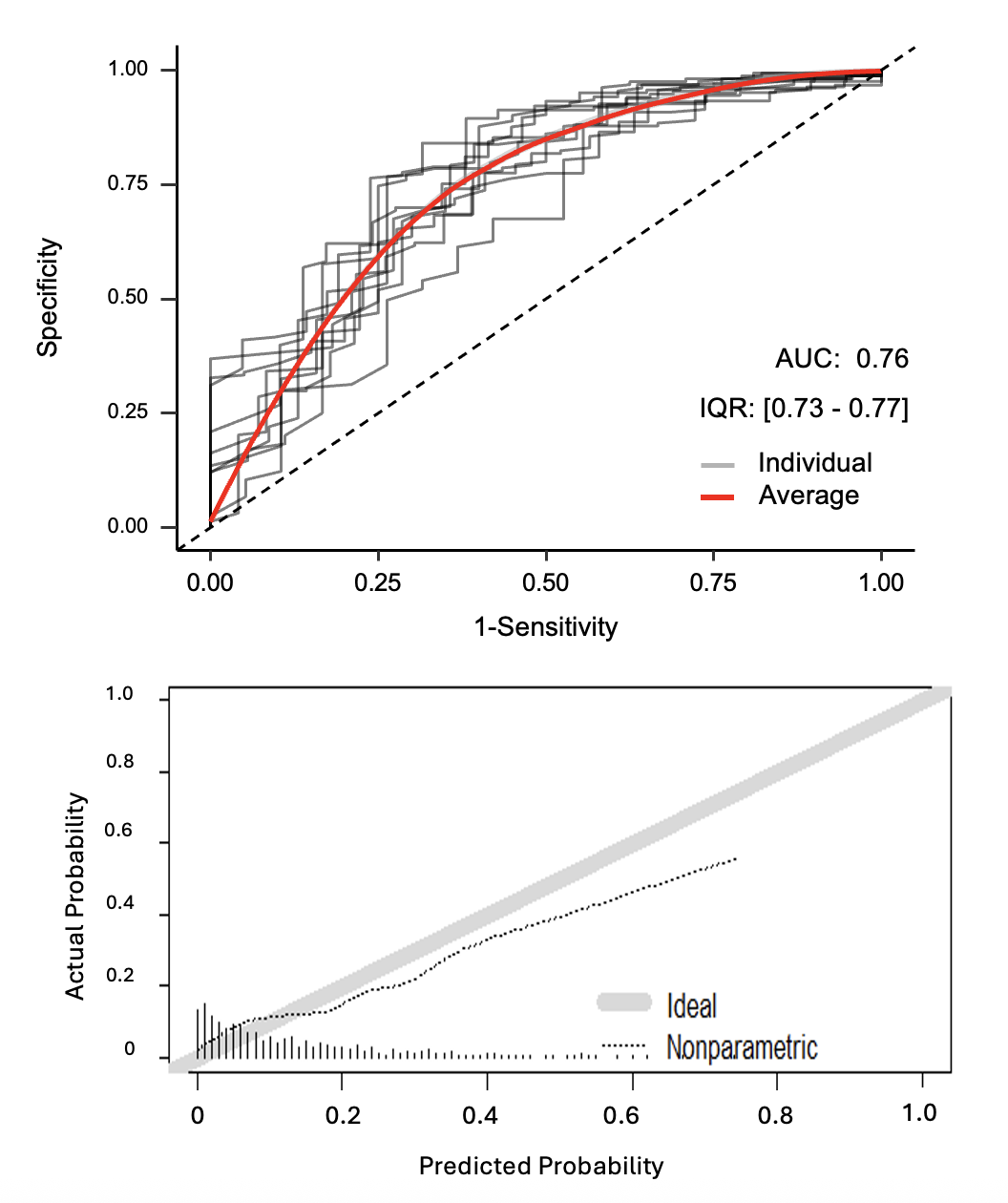


A calibration plot from 730 patient cohorts demonstrated well calibrated in low-risk patients. An AUC of 0.76 demonstrated fair discrimination in this cohort.

**Supplemental Figure S2 - Example of score calculation using the clinical risk prediction model**

**
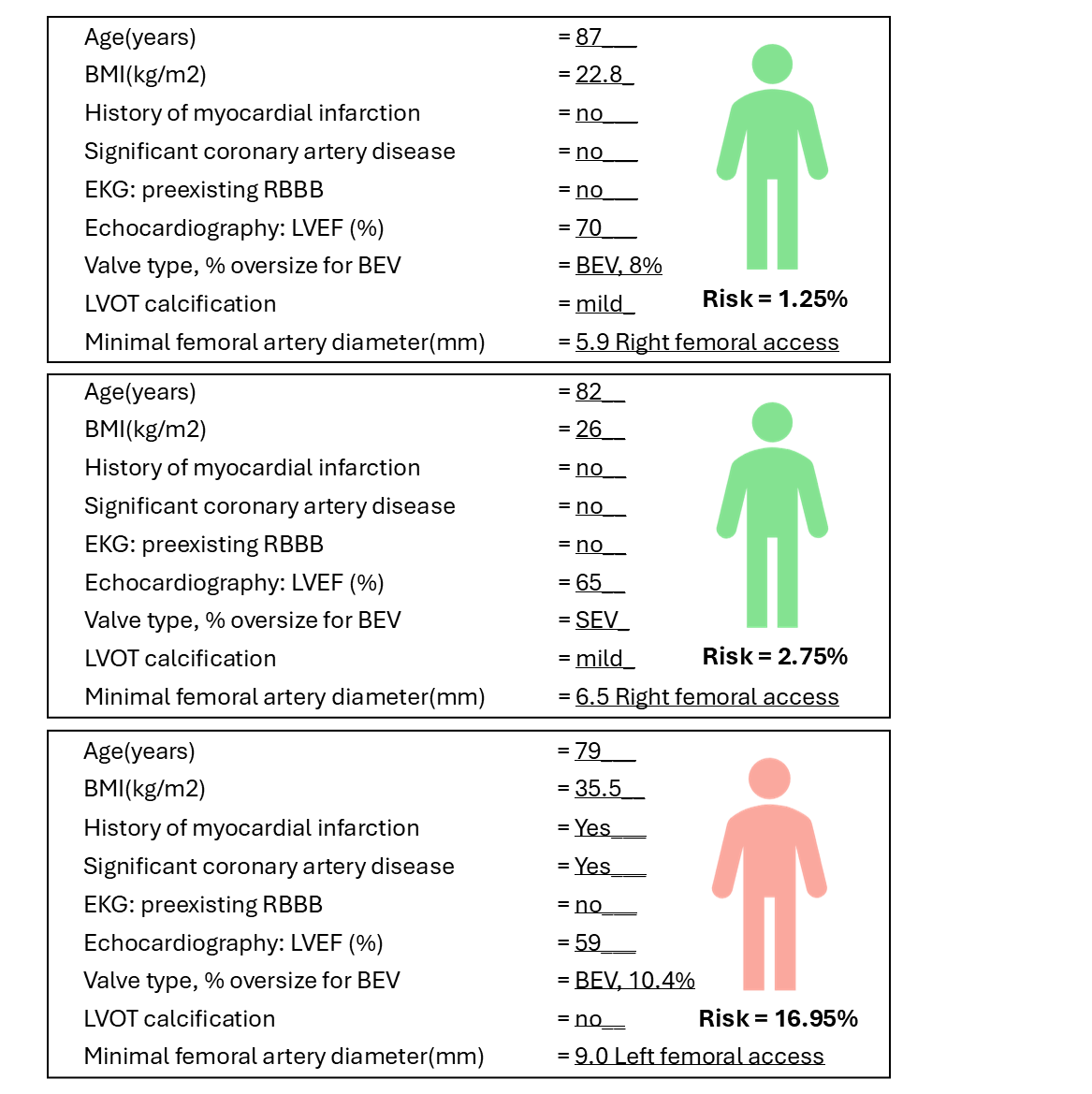
**

Risk score calculation was used to evaluate patient risk. BEV = balloon-expandable valve; BMI = body mass index; EKG = electrocardiography; LVEF = left ventricular ejection fraction; LVOT = left ventricular outflow tract; RBBB = right bundle branch block.
